# P04.11. Factors associated with willingness to participate in a yoga clinical trial among breast cancer survivors

**DOI:** 10.1186/1472-6882-12-S1-P281

**Published:** 2012-06-12

**Authors:** K Desai, M Galantino, S Li, M Boyle, B Hurtubise, J Mao

**Affiliations:** 1 University of Pennsylvania Health System, Philadelphia, USA; 2 Richard Stockton College of New Jersey, Galloway, USA

## Purpose

Due to an increased interest among researchers in studying the efficacy of yoga for symptom palliation among breast cancer survivors, understanding willingness to participation (WTP) in yoga clinical trials is critical for effective recruitment, especially for historically under-represented groups.

## Methods

We performed a cross-sectional survey study at an urban academic cancer centre among outpatient postmenopausal women with stage 0 to III breast cancer receiving adjuvant aromatase inhibitor (AI) therapy. Self-reported WTP in a yoga trial was the main outcome variable. Perceived barriers to WTP were collected along with sociodemographic and clinical variables. Multivariate logistic regression was used to identify factors associated with WTP.

## Results

Four hundred thirty-three patients participated, 357 (82.5%) whites, 63 (14.6%) blacks, seven (1.6 %) Asians, and six (1.3%) others. Of the participants, 261 (60.3%) reported WTP in a yoga clinical trial and 52 (12%) had used yoga since their breast cancer diagnosis. In multivariate analysis, higher likelihood of WTP was associated with younger age groups: < 55 years [adjusted odds ratio (AOR) 2.42, 95% Confidence Interval (CI) 1.32-4.44] and age 55-65 years (AOR 2.91, CI 1.32-6.41); having higher education: college education ( AOR 3.0, CI 1.64-5.5) and graduate or professional school (AOR 3.27 CI 1.70-6.32); and previous use of yoga (AOR 4.52, CI 1.66-12.22). Perceived barriers including ‘responsibilities at home’ (AOR 0.55, CI 0.33-0.91) and ‘didn’t want to be experimented on’ (AOR 0.51, CI 0.30-0.87) were associated with decreased WTP in a yoga trial. Unexpectedly, race/ethnicity was not associated with WTP in multivariate analyses.

## Conclusion

The majority of breast cancer survivors expressed WTP in yoga research; however, older age and lower education were associated with decreased likelihood to participate. Thus, thoughtful study design and tailored recruitment strategies are needed to minimize disparities in yoga trial participation, particularly those related to age and education.

